# Combined Effects of Physical Behavior Compositions and Psychosocial Resources on Perceived Exertion Among Eldercare Workers

**DOI:** 10.1093/annweh/wxaa079

**Published:** 2020-07-30

**Authors:** Leticia Bergamin Januario, Matthew L Stevens, Svend Erik Mathiassen, Andreas Holtermann, Kristina Karstad, David M Hallman

**Affiliations:** 1 Centre for Musculoskeletal Research, Department of Occupational Health Sciences and Psychology, University of Gävle, Gävle, Sweden; 2 Musculoskeletal Disorders and Physical Workload, National Research Centre for the Working Environment, Copenhagen, Denmark

**Keywords:** compositional data analysis, healthcare, physical exertion, psychosocial factors, triaxial accelerometers

## Abstract

**Objectives:**

High perceived physical exertion is common in eldercare workers and a strong predictor for impaired health. However, little is known on how physical behaviors at work associate with physical exertion in this group. The aim of this study was to determine the extent to which the composition of physical behaviors at work is associated with perceived physical exertion in nursing home eldercare workers, and the extent to which these associations are modified by psychosocial resources.

**Methods:**

Our population consisted of 399 female eldercare workers from 126 wards in 20 different nursing homes. We evaluated time spent in physical behaviors at work [sitting, standing still, light activities (LAs), and moderate to vigorous activities (MVAs)] using triaxial accelerometers worn, on average, for three working days. We accounted for inherent codependency between the behaviors using compositional data analysis. We used multilevel linear mixed regression models to determine associations between the behaviors and perceived exertion, measured on a numeric rating scale (0–10), and included interactions between each behavior and psychosocial resources (influence at work, social support, and quality of leadership) to determine a possible moderating effect of resources. Regression results were illustrated using isotemporal substitution.

**Results:**

Sitting was negatively (*β*: −0.64; *P* < 0.01) while MVA was positively (*β*: 0.95; *P* = 0.02) associated with perceived exertion. According to isotemporal substitution, replacing 30 min of MVA by sitting would, for an average worker, be associated with a decrease in physical exertion by −0.14 on the 0–10 scale. Job resources marginally moderated the association between LA and exertion. Thus, among workers with low influence and low social support, we found a positive association between LA and exertion, while that was not found for workers with medium or high influence and support (interactions for influence and support: *P* = 0.08 and *P* = 0.10).

**Conclusions:**

Our findings suggest that reallocating time from MVA to sitting can mitigate perceived physical exertion in eldercare workers. More time in LA increased physical exertion only for workers with low psychosocial resources, supporting a positive effect of a better psychosocial work environment in elderly care.

## Introduction

Eldercare is a women-dominated occupation with high levels of physical and psychosocial demands, typical of healthcare work in general (Gunnarsdottir *et al.*, 2004; Long *et al.*, 2012). Both physical and psychosocial demands are associated with musculoskeletal symptoms, sickness absence, and early retirement intentions among healthcare workers in nursing homes and hospitals (Andersen *et al.*, 2012a; Jensen *et al.*, 2012; Clausen *et al.*, 2014; Gold *et al.*, 2017), and these outcomes are strongly predicted by perceived physical exertion (Feng *et al.*, 2007; Andersen *et al.*, 2013, 2019; Burgel and Elshatarat, 2017). Also, eldercare workers perceive more physical exertion than workers in several other blue-collar occupations. For example, Karstad *et al.* (2018) reported an average level of perceived physical exertion among nursing home eldercare workers of 6.6 on a 0–10 scale, which is considerably higher than levels reported for construction workers (average 2.8) (Persson *et al.*, 2006), garment workers (average 3.7) (Wang *et al.*, 2009), and workers in agriculture (average 2.7) (Kolstrup, 2012).

Perceived physical exertion reflects the balance between physical demands and individual capacity (Andersen *et al.*, 2012a), and echoes a complex sensory experience integrating both physiological and psychological aspects of work. As such, it is considered a biopsychosocial measure (Borg, 1982; Koltyn, 2005; Borg and Kaijser, 2006). Identifying determinants of physical exertion in eldercare workers can, therefore, be an important step toward developing interventions preventing musculoskeletal symptoms and sickness absence in this occupational group (Feng *et al.*, 2007; Andersen *et al.*, 2012a).

Eldercare workers experience a number of potentially harmful physical behaviors at work, such as extensive standing and walking with limited opportunities to sit down and rest (Trinkoff *et al.*, 2001; Pelissier *et al.*, 2014; Chappel *et al.*, 2017). These behaviors likely contribute to determining the perceived physical exertion, but to our knowledge, no previous studies have evaluated associations between physical behaviors in eldercare workers and perceived exertion. Time spent in different physical behaviors in healthcare settings has commonly been assessed using self-reports (Nahm *et al.*, 2012; Perry *et al.*, 2015; Saridi *et al.*, 2019). However, self-reported physical behavior is known to be less accurate than objective measurements, and prone to differential bias (Prince *et al.*, 2008; Gupta *et al.*, 2018; Hallman *et al.*, 2019).

Time spent in any physical behavior is part of a finite period of time (e.g. a workday of approximately 8 h), and all behaviors during that time together form a temporal composition of the workday. If time spent in any one behavior at work is changed, time in at least one other behavior within that workday will inevitably also change. Data in a composition can be adequately analyzed using a customized approach known as compositional data analysis (CoDA) (Pedišić, 2014; Chastin *et al.*, 2015; Dumuid *et al.*, 2018, 2020; Gupta *et al.*, 2020). In CoDA, ratios are formed between parts of the composition, and these ratios are log-transformed, so that further data analysis can proceed according to standard statistical techniques. Thus, CoDA can handle the entire composition of time in different physical behaviors at work, replacing the ordinary procedure of addressing only one single behavior at a time.

Since perceived physical exertion is a biopsychosocial construct, it is important to realize that it can be affected by physical as well as psychosocial factors at work (Andersen *et al.*, 2012a; Januario *et al.*, 2019). Low demands, such as little quantitative demands and low work pace; and high resources, such as good influence and social support, have been associated with less perceived physical exertion among eldercare workers (Januario *et al.*, 2019). Psychosocial resources may even act by moderating negative effects of high demands, that is, they may have a ‘buffering effect’ as described in the Job Demand-Resources model (Demerouti *et al.*, 2001; Bakker and Demerouti, 2007).

Therefore, understanding the structure and extent of the relationships between perceived physical exertion, physical exposure at work, and psychosocial resources is an important step in developing interventions that can effectively reduce exertion, particularly in occupations with high physical and psychosocial demands, such as eldercare (Hauke *et al.*, 2011; Clausen *et al.*, 2014). This study aims at determining the extent to which the composition of physical behaviors at work is associated with perceived physical exertion among eldercare workers at nursing homes, and the extent to which these associations are modified by psychosocial resources at work.

## Methods

### Design and study population

We used baseline data obtained from the Danish Observational Study of Eldercare work and musculoskeletal disorderS (DOSES) (Karstad *et al.*, 2018). DOSES is a multilevel study based on data nested in three hierarchical levels (nursing homes, wards, and workers). Details of the study protocol have been described in Karstad *et al.* (2018). The Danish Data Protection Agency and the regional Ethics Committee in Copenhagen, Denmark (H-4-2013-028) approved the study and all participants provided their written informed consent to participate.

Briefly, data were collected between September 2013 and December 2014 from 553 eldercare workers employed in 126 wards distributed across 20 nursing homes located in the greater Copenhagen area. Workers were included if they were aged between 18 and 65 years, employed for more than 15 h per week, worked on day, evening, or changing shifts (but not night), and spent at least 25% of their working time on tasks related to the actual care of residents. These caring activities involved, for example, lifting, repositioning, and turning the resident with or without assistance; dressing the resident; and moving the resident in a portable chair. In Denmark, most eldercare workers have an education as social and health service helpers (SHS helpers) with 14 months of training, or as social and health service aides (SHS aides) with an additional 6 months of training, allowing them to work in hospitals and psychiatry (Karstad *et al.*, 2018).

In this study, we used data from self-administered questionnaires, accelerometer-based recordings of physical behaviors, and measured height and body weight. In the initial sample, 419 participants provided all the data needed for the present study. However, as only 5% of the original sample were men, we restricted our sample to women in order to eliminate possible confounding by gender. Thus, the final population for this study consisted of 399 female workers.

### Measurements

#### Perceived physical exertion (outcome)

We measured perceived physical exertion using a single-item question, i.e. ‘How physically hard do you perceive your current work to be?’ (free translation from Danish to English), answered on a numeric scale ranging from 0 (‘not hard’) to 10 (‘extremely hard’) (Borg and Kaijser, 2006).

#### Accelerometer measurements of physical behavior (exposure)

We assessed physical behaviors at work using data from two triaxial ActiGraph GT3X+ accelerometers (Actigraph, Pensacola, FL, USA) placed on the upper back and right thigh. All participants wore the accelerometers without interruption for at least four consecutive days, including at least two workdays. We identified working, leisure, and sleeping hours from a diary filled in by the participants. For the present study, we considered only accelerometer data from working hours, and only if they included at least 4 h of work, or at least 75% of the shift (Gupta *et al.*, 2016).

We used the validated custom-made MATLAB software Acti4 for identifying behaviors on basis of the accelerometer data (Skotte *et al.*, 2014). For this study, we assessed time in sitting, standing still, moving (i.e. occasional movements in an upright position), slow walking (defined as a cadence of less than 100 steps per minute), fast walking (defined as a cadence of more than 100 steps per minute), climbing stairs, running, and cycling (Ingebrigtsen *et al.*, 2013; Skotte *et al.*, 2014). To detect walking or running, the epoch length should be more than 2 s (as default of the Acti4 software setting). The step cadence was calculated second by second in all epochs in walking, then averaged during work hours, quantified in steps per minute and classified in slow and fast walking. These behaviors were then merged into four categories: (i) sitting, (ii) standing still, (iii) light physical activities (LA: moving and slow walking), and (iv) moderate to vigorous physical activities (MVAs: fast walking, climbing stairs, running, and cycling). We calculated the mean of each behavior across days for each participant in terms of both minutes spent per day and percentage of worktime, and then calculated the arithmetic means, SDs and ranges of these individual means across the population as summary measures of behavior.

#### Psychosocial resources (moderators)

Psychosocial job resources—which we considered as possible moderators of the association between physical behaviors and perceived physical exertion—were measured in three dimensions, i.e. influence at work, social support, and quality of leadership, using the Copenhagen Psychosocial Questionnaire (COPSOQ) (Pejtersen *et al.*, 2010).

Workers rated influence at work (two items—IN1 and IN3; cf. Pejtersen *et al.*, 2010), social support (three items—SC1, SC2, and SC3), and quality of leadership (four items—QL1, QL2, QL3, and QL4) using 5-point Likert scales. We converted answers to 0–100 scores before averaging items within each dimension, whereby higher scores express better psychosocial working conditions (Pejtersen *et al.*, 2010). For interpretation purposes, we categorized, for each dimension, the participants into tertiles (low, middle, and upper) based on their COPSOQ results.

#### Confounders

We considered a number of confounder candidates, based on previous literature and theoretical assumptions of the possible associations with perceived physical exertion, physical behavior at work, and psychosocial resources. These confounder candidates included age (years), body mass index (BMI, kg m^−2^; calculated from the measured height and body weight), job title (SHS aide, SHS helper, or other type), shift (day, evening, or changing shifts), leisure time physical activity [accelerometer-based moderate to vigorous activities (MVAs) performed during leisure], type of ward (somatic, dementia, or other type of ward), and staffing ratio (number of eldercare workers in the ward divided by the number of residents).

### Data transformation (CoDA)

We described and analyzed the physical behaviors using a CoDA procedure, in which the absolute values of the four behaviors (minutes of sitting, standing, LA, and MVA) were transformed into sets of three log-ratios.

As an example, the ILR set for sitting was:

$$\begin{array}{l} ILR_{sit1}=\sqrt{\frac{3}{4}}ln\frac{time_{sit}}{\sqrt[{3}]{time_{stand}\times time_{LA}\times time_{MVA}}} \end{array}$$ (1)

$$\begin{array}{l} ILR_{sit2}=\sqrt{\frac{2}{3}}ln\frac{time_{stand}}{\sqrt[{2}]{time_{LA}\times time_{MVA}}} \end{array}$$ (2)

$$\begin{array}{l} ILR_{sit3}=\sqrt{\frac{1}{2}}ln\frac{time_{LA}}{time_{MVA}} \end{array}$$ (3)

Time_sit_, time_stand_, time_LA_, and time_MVA_ in these equations represent the percentage of time spent in each behavior during the workday. ILR_sit1_ represents the ratio of sitting time to time spent in all other behaviors (nonsitting), expressed as a geometric average. ILR_sit2_ represents the ratio of standing time to time spent in light and in MVAs, within nonsitting time. ILR_sit3_ expresses the ratio of time in LA to time in MVA.

We calculated four such sets of ILRs by rearranging (or ‘rotating’; Dumuid *et al.*, 2018) each behavior into the numerator position of ILR_1_ (equation (1)), and subsequently adjusting the remaining equations. The reason for developing four sets of ILRs (ILR_sit_, ILR_stand_, ILR_LA_, and ILR_MVA_) is that in the interpretation of the eventual statistical analysis results, only effect estimates for the first ILR (i.e. ILR_1_) is directly interpretable (Pedišić *et al.*, 2017).

Thus, in the eventual statistical models, effects associated with ILR_sit1_ represent the effect of sitting time relative to time spent in all nonsitting behaviors; ILR_stand1_ the effect of standing time relative to time spent in all nonstanding behaviors; ILR_LA1_ the effect of time spent in light activities (LAs) relative to time in all non-LA behaviors; and ILR_MVA1_ the effect of time in MVAs relative to time spent in all non-MVA behaviors.

### Statistical analysis

We adopted a multilevel linear mixed regression model (Heck *et al.*, 2013) to account for the nested structure of the data (workers within wards within nursing homes) by using wards and nursing homes as random effects. We used the Statistical Package for the Social Sciences (SPSS, v 24.0, IBM, Armonk, NY, USA) for the main analyses and R/RStudio (with packages ‘compositions’, ‘robcompositions’, ‘BSDA’, ‘lme4’, and the ‘tidyverse’ suite of packages) for further examination of the CoDA results.

In theory, the confounder candidates could act as moderators on the association between physical behavior at work and perceived physical exertion, rather than as confounders *per se*. Therefore, all confounder candidates were tested, one after the other, for a moderation effect, by assessing their interactions with each behavior (sitting, standing, LA, and MVA) in determining the outcome, using a linear mixed model. These analyses showed that none of the confounder candidates had a significant interaction (all *P* > 0.1) with any ILR_1_. We therefore disqualified them as moderators and treated them as candidates for having confounding effects.

Next, we examined correlations between confounder candidates, exposures (ILR_1_ for each behavior), and outcome (perceived physical exertion). We used Pearson’s and Spearman’s correlations to evaluate continuous and categorical variables, respectively. If the confounder candidate was correlated with both outcome and exposure (*P* ≤ 0.1), it was accepted as a potential confounder. Based on this criterion, we eventually included age, type of ward, and staff ratio as confounders in the adjusted models.

To examine associations between behaviors and perceived exertion, we performed four sets of multilevel linear mixed models using the four ILR_1_, one after the other, as the predictor and physical exertion as the outcome. First (model 1), we determined the unadjusted association between the physical behavior (e.g. ILR_sit1_) and perceived physical exertion, while including the remaining ILRs (in the example, ILR_sit2_ and ILR_sit3_) as covariates to adjust for the remaining behaviors (Chastin *et al.*, 2015; Dumuid *et al.*, 2019). Second (model 2), we adjusted model 1 by including the confounders age, type of ward, and staff ratio. Third (model 3), we added psychosocial resources (influence, social support, and quality of leadership) to model 1 as fixed effects, as well as their interaction with the physical behavior composition, to determine the moderating effect of psychosocial resources on the associations between physical behavior and perceived physical exertion. Finally (model 4), we tested the adjusted moderating effect of psychosocial resources by including the confounders age, type of ward, and staff ratio in model 3. These four multilevel linear mixed models were resolved for all four sets of ILRs, i.e. with ILR_sit1_, ILR_stand1_, ILR_LA1_, and ILR_MVA1_ as the primary predictor, respectively. Effect estimates were expressed as beta (*β*) coefficients with 95% confidence intervals (CIs) and corresponding *P*-values, <0.05 indicating statistical significance.

### Isotemporal substitutions

Even though it is possible to interpret the directionality of effect estimates, *β*, in a compositional space where independent variables are expressed as ILR coordinates, numeric effect sizes in terms of minutes or percentage time cannot be easily ascertained. For this reason, we used isotemporal substitutions to illustrate the estimated perceived physical exertion when reallocating time between behaviors (Dumuid *et al.*, 2019). We did this initially by progressively increasing time (15, 30, 45, and 60 min) in one specific behavior, while correspondingly and proportionally decreasing time spent in the three remaining behaviors, i.e. a one-to-many method, that illustrates a general effect of changing that single behavior (Stevens *et al.*, 2020). If we found significant associations between at least two physical behaviors and exertion in model 1, we also reallocated time between those behaviors (leaving time spent in other behaviors unchanged), i.e. a one-to-one method, as a means to illustrate more practically the effects of replacing time in a specific behavior with another (Stevens *et al.*, 2020). We conducted these substitutions according to procedures described by Dumuid *et al.* (2019).

## Results

The study population is described in Table 1. The workers reported, on average, a perceived physical exertion at work of 6.9 (SD 1.9) on the 0–10 scale. On average, they wore the accelerometers for 3.0 workdays, including, on average, 6.9 (SD 1.4) h of work per day. The COPSOQ scores (scale from 0 = low to 100 = high) were, on average, 57.2 (SD 19.1) for influence at work, 71.8 (SD 14.8) for social support, and 60.3 (SD 17.2) for quality of leadership.

**Table 1.** General characteristics of the study population (*n* = 399).

Variables	*n* (%)	Mean (SD)	Range
Age (years)^a^	—	46.2 (10.5)	21.0–71.0
BMI (kg m^−2^)^a^	—	26.5 (5.4)	14.6–47.4
Staff ratio (worker/resident)^b^	—	0.5 (0.1)	0.2–0.8
Perceived physical exertion (scale 0–10)^a^	—	6.9 (1.9)	0.0–10.0
LTPA (%time in MVA)^c^	—	10.0 (4.8)	2.2–35.7
Occupational behavior (min day^−1^)^c^			
Sitting	—	155.8 (61.6)	7.7–383.2
Standing	—	135.2 (41.5)	4.8–251.3
LA	—	67.1 (23.0)	1.7–139.7
MVA	—	58.5 (19.9)	2.4–108.3
Occupational behavior (% worktime)^c^			
Sitting	—	37.4 (12.5)	8.8–81.0
Standing	—	32.5 (7.8)	8.8–54.6
LA	—	16.1 (4.8)	3.1–33.3
MVA	—	14.0 (3.9)	3.9–28.5
Job title^a^			
SHS helper	171 (42.9)	—	—
SHS aide	164 (41.1)	—	—
Other	64 (16.0)	—	—
Work shift^a^			
Day	230 (57.6)	—	—
Changing	78 (19.5)	—	—
Evening	91 (22.8)	—	—
Type of ward^b^			
Somatic unit	291 (74.4)	—	—
Dementia unit	79 (20.2)	—	—
Other unit	21 (5.4)	—	—
Psychosocial resources (on a 0–100 scale, categorized in tertiles)^a^			
Influence—lower	91 (22.8)	30.6 (10.1)	28.5–32.7
Influence—middle	183 (45.9)	55.7 (6.2)	54.8–56.6
Influence—upper	125 (31.3)	78.7 (7.6)	77.4–80.0
Social support—lower	100 (25.1)	52.1 (7.6)	50.6–53.6
Social support—middle	170 (42.6)	70.9 (4.2)	70.3–71.6
Social support—upper	129 (32.3)	88.3 (5.6)	87.3–89.3
Quality of leadership—lower	133 (33.6)	41.5 (10.3)	39.7–43.3
Quality of leadership—middle	112 (28.3)	59.8 (3.1)	59.2–60.3
Quality of leadership—upper	151 (38.1)	77.2 (8.9)	75.8–78.7

BMI, body mass index; LTPA, leisure time physical activity; SHS, social and health service.

^*a*^Workers’ self-report.

^*b*^Information provided by team managers.

^*c*^Accelerometer data.

**Figure 1.**
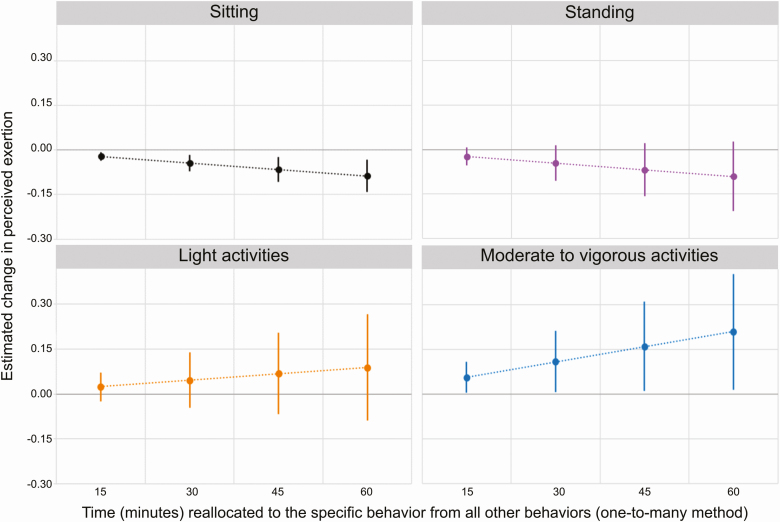
Estimated change in perceived physical exertion when reallocating time to a specific behavior from all other behaviors (one-to-many method), relative to the average composition of the sample. Diagrams show the estimated change in exertion, with 95% CI, from the mean score (6.9 on the 0–10 scale) of all participants, according to model 2.

Time sitting relative to nonsitting was negatively associated with perceived physical exertion, both in model 1 (*β* for ILR_sit1_: −0.64, 95% CI: [−1.04; −0.24]) and in model 2 (*β* for ILR_sit1_: −0.58, 95% CI: [−0.98; −0.17]) (Table 2). According to isotemporal substitution, a worker having 30 min more of sitting than the average worker, at the expense of time in all other behaviors (the one-to-many reallocation), would be expected to report a slightly lower physical exertion than the average worker, i.e. by −0.04 on the 0–10 scale; 95% CI: [−0.07; −0.02] (Fig. 1).

**Table 2.** Compositional analyses of associations between occupational behaviors (sitting, standing, LAs, and MVAs), and perceived physical exertion.

Behavior	Model 1^a^			Model 2^b^		
	*β*	95% CI	*P*	*β*	95% CI	*P*
ILR_sit1_	**−0.64**	**−1.04; −0.24**	**<0.01**	**−0.58**	**−0.98; −0.17**	**0.01**
ILR_stand1_	**−**0.56	**−**1.40; 0.27	0.19	**−**0.57	**−**1.40; 0.27	0.18
ILR_LA1_	0.24	**−**0.55; 1.04	0.55	0.36	**−**0.46; 1.18	0.39
ILR_MVA1_	**0.95**	**0.15; 1.76**	**0.02**	0.78	**−**0.04; 1.60	0.06

ILR, isometric log-ratio; *β*, effect estimates; 95% CI (lower bound; upper bound). Bold values represent associations with *β*-values differing from zero at a *P* < 0.05 level.

^*a*^Model 1: unadjusted, including ILR_2_ and ILR_3_.

^*b*^Model 2: model 1, further adjusted by age, type of ward, and staff ratio.

**Figure 2.**
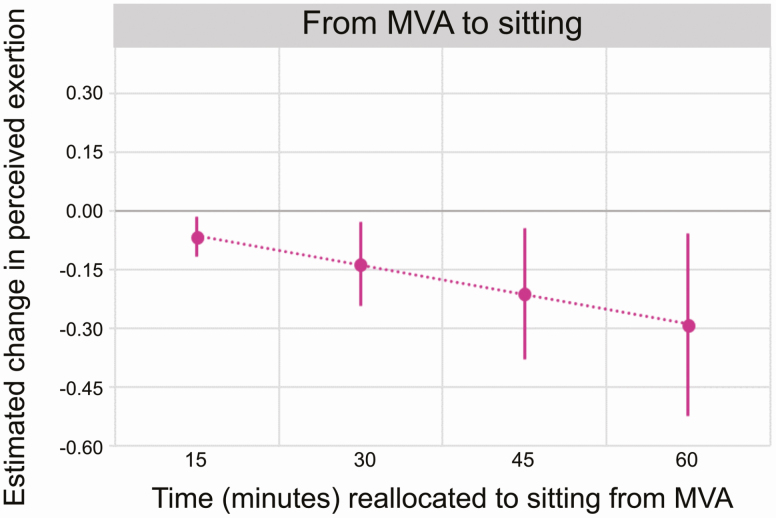
Estimated change in perceived physical exertion when reallocating time to sitting from MVAs (one-to-one method), relative to the average composition of the sample. Diagram shows the estimated change in exertion, with 95% CI, from the mean score (6.9 on the 0–10 scale) of all participants, according to model 2.

Time in MVA relative to non-MVA was positively associated with perceived physical exertion (*β* for ILR_MVA1_: 0.95, 95% CI: [0.15; 1.76]) (Table 2), although this association was weaker after further adjustment in model 2 (*β* for ILR_MVA1_: 0.78, 95% CI: [−0.04; 1.60]). A worker with 30 min more MVA than the average (at the expense of all other behaviors, i.e. a one-to-many reallocation), would be expected to experience a larger physical exertion than the average worker (increase by 0.11; 95% CI: [0.01; 0.21] on the 0–10 scale) (Fig. 1). Standing and LA showed negative and positive associations with perceived physical exertion, respectively, but the estimated effects were uncertain with CIs overlapping zero (Table 2). The one-to-one isotemporal substitution method showed that reallocating 30 min from MVA to sitting led to a reduction in estimated physical exertion of −0.14 (95% CI: [−0.24; −0.03]) units (Fig. 2).

**Figure 3.**
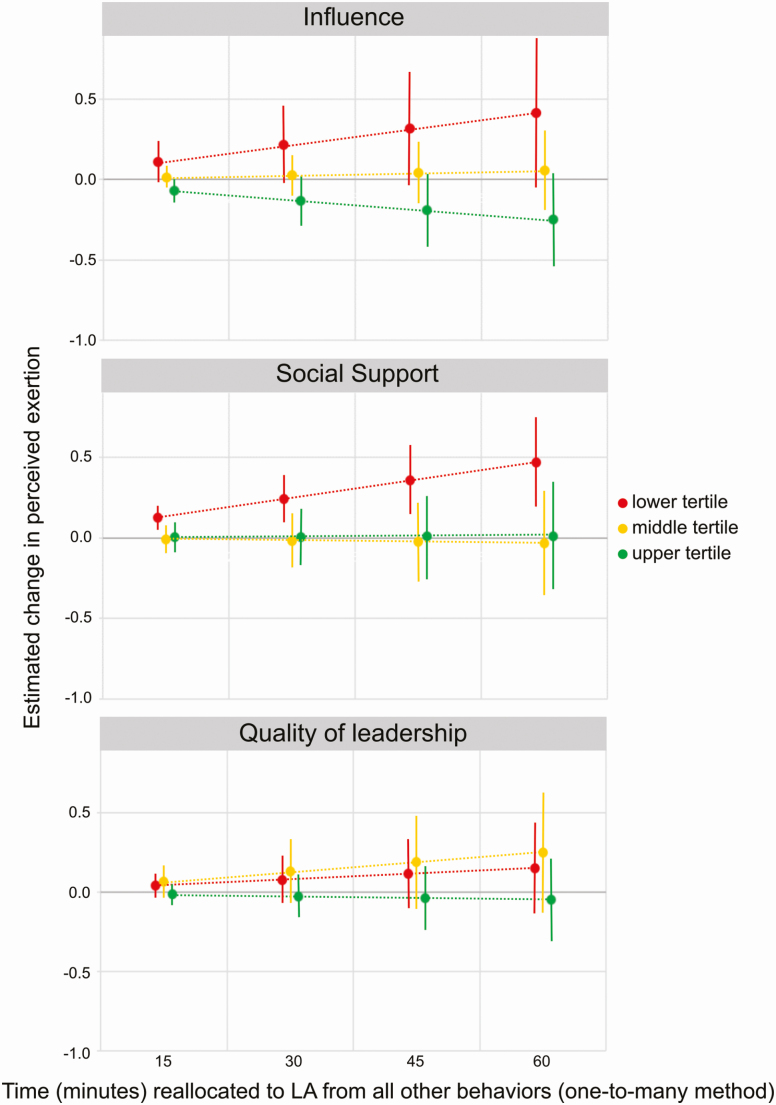
Moderating effects of psychosocial resources (stratified by lower, middle, and upper tertile), illustrated by the estimated change in perceived physical exertion when reallocating time to LAs from all other behaviors (one-to-many method), relative to the average composition of the sample. Diagrams show the estimated change in exertion, with 95% CI, from the mean score (6.9 on the 0–10 scale) of all participants.

Neither influence at work, social support, nor quality of leadership showed statistically significant interactions with sitting, standing, or MVA. However, we found a marginal interaction between LA and influence at work (model 3, *β*: −0.04, 95% CI: [−0.08; 0.00], *P*: 0.08; model 4, *β*: −0.04, 95% CI: [−0.08; 0.00], *P*: 0.06). As an example, reallocating 30 min to LA (one-to-many method) was associated with an increase of 0.22 (95% CI: [−0.02; 0.46]) in perceived physical exertion for the average worker with low influence at work, an increase of 0.03 (95% CI: [−0.10; 0.16]) for middle influence, and a decrease by −0.13 (95% CI: [−0.29; 0.02]) for high influence (Fig. 3).

Social support also had a marginal interaction with LA in predicting perceived physical exertion (model 3, *β*: −0.04, 95% CI: [−0.10; 0.01], *P*: 0.10; model 4, *β*: −0.04, 95% CI: [−0.10; 0.01], *P*: 0.10). Reallocating 30 min to LA (one-to-many method) was associated with an increase of 0.24 (95% CI: [0.10; 0.39]) in perceived physical exertion for an average worker with low social support, while a similar reallocation did not significantly change the estimated perceived exertion among workers with middle or high social support (difference of −0.02; 95% CI [−0.19; 0.15] and 0.01; 95% CI [−0.17; 0.18]) (Fig. 3).

We did not find any notable interaction effect for quality of leadership and LA in predicting perceived physical exertion (model 3, *β*: 0.00, 95% CI: [−0.05; 0.05], *P*: 0.93; model 4, *β*: 0.00, 95% CI: [−0.06; 0.04], *P*: 0.75).

## Discussion

This study examined associations between physical behaviors at work and perceived physical exertion among eldercare workers, as well as the moderating effects of psychosocial resources on these associations. We found that more time sitting and standing at work were associated with lower perceived physical exertion. In contrast, more time spent in LAs and MVAs were associated with more exertion. Furthermore, influence and social support marginally modified the association between LAs and perceived physical exertion. Perceived physical exertion was higher among workers spending a certain proportion of time in LAs if they had low influence and/or low social support than if they had middle or high psychosocial resources.

### Composition of behaviors and perceived physical exertion

We found that time in sitting relative to time in all nonsitting behaviors was negatively associated with perceived physical exertion, and that 30 min more sitting, at the expense of time in other behaviors, was estimated to decrease perceived physical exertion by 0.04 units, i.e. a small, but significant effect. Previous studies using objective measurements in blue-collar workers have also showed the potential benefits of increasing time sitting in physically demanding occupations (Gupta *et al.*, 2016; Hallman *et al.*, 2016a; Korshøj *et al.*, 2018). Likely, more time sitting allows the average eldercare workers, who sit less than the average worker in many other occupations (Gilson *et al.*, 2019; Jørgensen *et al.*, 2019), to recover from physically demanding tasks. Energy expenditure is less in sitting than in most other wake behaviors (Saeidifard *et al.*, 2018), and thus more sitting may prevent extensive accumulation of exertion during periods with demanding tasks (Korshøj *et al.*, 2018). When the eldercare workers are not seated they are likely exposed to tasks that are more strenuous, such as lifting, turning, and supporting the resident, than when sitting, such as while feeding the elderly, updating medical charts, or watching television (da Costa and Vieira, 2010; Korshøj *et al.*, 2018).

Standing was also negatively associated with perceived exertion, with an effect size estimate similar to that of sitting, yet more uncertain and thus not statistically significant. The greater uncertainty of this association might be explained by standing being a more heterogeneous proxy for other, concurrent exposures than sitting. For example, standing talking next to a resident will inevitably imply a lower physical load than standing while holding or lifting a resident.

We found that physical activity at work, whether classified as light (LA: walking slow and moving) or moderate to vigorous (MVA: walking fast, running, climbing stairs, and cycling), was positively associated with perceived physical exertion. Since exertion may be a proxy for exposures negative to health, this corroborates previous studies indicating that high levels of occupational physical activity are associated with compromised health, particularly among blue-collar (Holtermann *et al.*, 2018; Skarpsno *et al.*, 2018) and healthcare workers (Allesøe *et al.*, 2015). High levels of physical activity at work increase energy demands and, thus, physiological strain among healthcare workers (Chen *et al.*, 2011; Chappel *et al.*, 2017). Likely, these effects are particularly pronounced if such activities are performed for long periods without interruption, if the workers do not have adequate breaks, and if recovery between shifts is insufficient (Chen *et al.*, 2011; Chappel *et al.*, 2017).

Our results showed that more time sitting was associated with less exertion, and that more time in MVA can be expected to increase exertion, at least in eldercare workers. However, we emphasize that our results, as illustrated using isotemporal substitutions, suggested that the effects of changing time in sitting or MVA on perceived exertion were very small, both in the one-to-many and one-to-one substitutions. However, even small changes in exertion may be of clinical importance in this population, reporting a high average exertion, i.e. 6.9 on a 0–10 scale. Previous studies have shown that strenuous physical exertion [>6 on a 0–10 scale (Borg, 1982; Borg and Kaijser, 2006)] increases the risk for long term sickness absence and chronic low back pain (Andersen *et al.*, 2012b, 2013), and has strong associations with muscle fatigue (≥8) (Cote *et al.*, 2008). Therefore, interventions leading to reduced physical exertion in the present population would likely result in less musculoskeletal symptoms, sickness absence, and early retirement intentions (Feng *et al.*, 2007; Wang *et al.*, 2009; Andersen *et al.*, 2013, 2019; Burgel and Elshatarat, 2017).

Notably, in the isotemporal substitutions, effects on perceived exertion are estimated for a worker with the average composition of physical behaviors, while in our population, sitting differed widely between workers, ranging from 9 to 81% working time. Thus, the effects of changing behaviors will likely differ between workers, and interventions should consider that some eldercare workers are more active at work and have restricted time for sitting while others have more opportunities to sit down. In addition, the recovery effect of sitting likely differ between shorter and long bouts of sitting (Gupta *et al.*, 2016; Hallman *et al.*, 2016b), and even depends on the behavior, e.g. MVA preceding sitting, and how long that was performed. Also, it is important to consider that we did not distinguish sitting during breaks from sitting while performing a work task, which can entail important differences in terms of physical demands (Blangsted *et al.*, 2004; Chaikumarn *et al.*, 2018). In summary, effects of interventions promoting sitting in the present population may depend on the extent and temporal distribution of behaviors, including what activities workers do while sitting, which, in turn, likely relates to organizational factors at the wards.

### Moderating effect of psychosocial resources

We found that influence at work and social support had a buffering effect on perceived physical exertion when the eldercare workers were performing LAs. Thus, workers with low influence and low social support were found to face larger increases in perceived physical exertion when spending more time in LAs (Fig. 2), in comparison with those classified as having middle or upper levels of influence and social support.

While the eldercare workers having lower and middle influence showed increases in perceived physical exertion when doing LAs, exertion decreased for those with higher influence at work (i.e. the upper tertile). Possibly, eldercare workers with high influence (i.e. those more able to make decisions and more responsible for their tasks) have better opportunities to arrange their tasks according to their own discretion, rather than having to follow schedules set by others. Thus, they may be more free to distribute physically demanding tasks across their shift (Fjell *et al.*, 2007; Xanthopoulou *et al.*, 2007; Clays *et al.*, 2016; Vogel *et al.*, 2017), which, in turn, may lead to a lower perceived exertion (Januario *et al.*, 2019).

Social support may attenuate the perceived effects of physically demanding work tasks, or it may represent a work environment where physically demanding tasks can be better adjusted to the individual’s physical capacity (Clays *et al.*, 2016) because colleague workers can help their peers during physically demanding tasks (Andersen *et al.*, 2012a). A study evaluating the effects of social support on the performance of a strenuous exercise challenge showed that perceived physical exertion was less among participants experiencing social support than among participants with no social support (Davis and Cohen, 2018). In this sense, workers who trust and rely on their peers may experience a better self-efficacy when performing their work tasks than workers with low social support, and this may lead to less perceived physical exertion when performing LAs (Christensen *et al.*, 2006; Schnall *et al.*, 2008).

Our results can help practitioners decrease perceived exertion among eldercare workers in suggesting that interventions should focus on reducing light and moderate to vigorous physical activity at work. However, some occupational settings do not allow substantially changes in tasks and activities conducted during a working day, and thus opportunities to change behaviors may be limited. In such settings, promoting a work environment with good psychosocial resources may be an alternative way to decrease perceived physical exertion among the workers.

### Methodological considerations

The main strengths of this study were that physical behaviors at work were recorded using objective measurements, and that we used CoDA to examine the effects of different behaviors on perceived exertion. To our knowledge, this is the first study to apply CoDA in a study assessing associations between objectively measured physical behaviors at work and perceived physical exertion. CoDA opens for an understanding of how the entire composition of behaviors during work is associated with physical exertion, while also accounting for the high collinearity between behaviors. Traditional analysis cannot address this inherent collinearity of physical behavior data, which may lead to misleading results (Dumuid *et al.*, 2018).

However, the cross-sectional design of the study precludes any strong conclusions as to the causal effects of behaviors on perceived exertion. Also, we only considered the total time spent in the different behaviors, not the distribution of time within each behavior, which likely modify the effect of that behavior on exertion and health (Gupta *et al.*, 2016; Hallman *et al.*, 2016b). However, addressing time patterns was not within the scope of the present study. We encourage further research into the effects of timing of tasks and activities—i.e. variation (Mathiassen, 2006)—on perceived exertion, including determinants of this variation at the organizational and individual level. In identifying physical behaviors on basis of the accelerometer recordings, we could not associate perceived exertion with metabolic parameters, such as cardiovascular responses (Jakobsen *et al.*, 2014; Jørgensen *et al.*, 2019). Thus, we encourage future studies evaluating the effects of physical behaviors to also assess biomechanical and physiologic characteristics of each behavior.

## Conclusion

When examining the composition of objectively measured physical behaviors among eldercare workers, we found that more time spent sitting and standing were associated with less perceived physical exertion, while more time in light and moderate to vigorous physical activities were associated with more perceived physical exertion. Psychosocial resources, i.e. influence and social support, were found to modify the effect of LAs, i.e. walking slow and moving, on perceived exertion. Our results support the importance of better psychosocial work environment in elderly care, even in the context of alleviating perceived exertion.

## Funding

AFA Insurance (grant number 180076) funded this work.
